# Editorial: The adipose tissue microenvironment in cancer: Molecular mechanisms and targets for treatment

**DOI:** 10.3389/fcell.2022.954645

**Published:** 2022-10-19

**Authors:** Yunlong Yang

**Affiliations:** Department of Cellular and Genetic Medicine, School of Basic Medical Sciences, Fudan University, Shanghai, China

**Keywords:** adipocyte, tumor microenvironment, host cells, metabolism, cancer-associated complications

Adipose tissue is one of the major components distributed throughout the human body and is considered as tissues that actively participates in metabolic and endocrine homeostasis. Cancer-associated adipocytes (CAAs) are commonly seen in tumors that originate from adipocyte-rich organs, which include breast cancer, ovarian cancer, colorectal cancer, and pancreatic cancers. Though CAAs were originally thought to be merely a passive, neutral, and terminally differentiated cell type during tumor progression, interest in them re-surged after they were identified to have multiple abilities to promote tumor growth, invasion, and metastasis. Recent evidence shows that CAAs respond to cancer cell-derived paracrine signaling to provide various adipokines, growth factors, as well as metabolic substrates for enhancing tumor cell proliferation and invasion (Quail and Dannenberg, 2019; Hoy et al., 2017). In addition to direct crosstalk between CAA and tumor cells, CAA participates in TME remodeling by altering the phenotype of other host cell types or by the phenotypic transition to fibroblast-like cells (Bochet et al., 2013). The remodeled TME in turn facilitates tumor growth, invasion, and drug resistance (Song et al., 2020; Iwamoto et al., 2018; Sheng et al., 2017). This indirect tumor-promoting effect of CAA has not been fully understood.

Other than adipocytes in the tumor, distant adipose depots outside the TME restlessly communicate with tumor tissue. Factors and metabolites released from the TME influence the distant tissues and organs, hence inducing cancer-associated complications. Cancer-associated cachexia (CAC) is the most critical cancer-associated complication and is estimated to be the cause of death in 1/4 of cancer patients (Baracos et al., 2018). Conversely, distant adipose depots provide metabolites, endocrine factors, and exosomes to regulate tumor development (Jafari et al., 2021). Together, adipocyte-tumor cell crosstalk not only happens in the tumor microenvironment, but exists in the tumor macroenvironment (Figure 1), which includes the tumor-associated systemic pathological interactions (Yang and Cao, 2022); Al-Zhoughbi et al., 2014). The difference between these two types of crosstalks is that the former is paracrine or autocrine, while the latter is largely endocrine. Here in this topic, we gathered original research and reviews to describe the mechanisms of adipocyte-tumor cell crosstalk in microenvironment and macroenvironment, and also highlight potential targets for regulating this unique cell-cell interaction.

**FIGURE 1:**
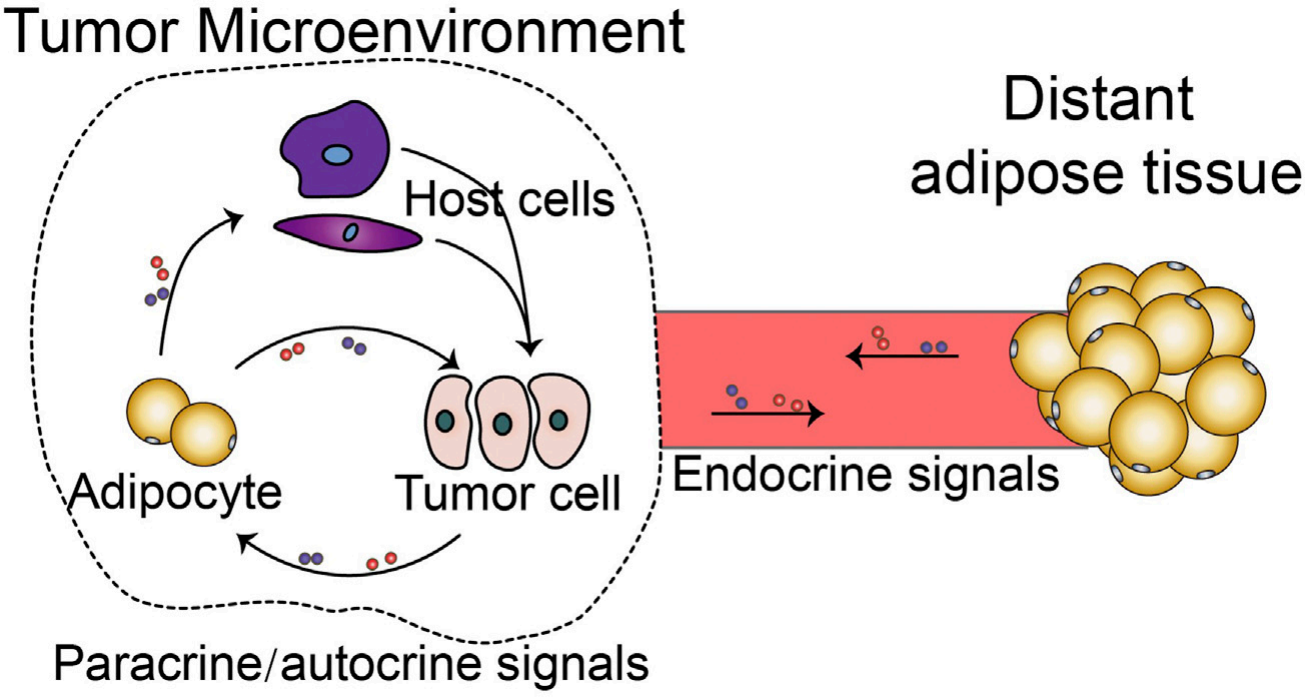
Adipocyte-tumor cell crosstalk in tumor microenvironment and tumor macroenvironment. (In the TME, CAA interacts with tumor cells *via* autocrine and paracrine signalings. Additionally, CAA indirectly promotes tumor cells *via* various host cell types in the TME. In contrast, distant adipose tissue systemically interacts with tumor cells or host cells *via* endocrine signalings).

Among various tumor types, breast cancer is the most well-known cancer that is closely associated with adipocytes. Here, Miran et al. performed a small clinical study in which human adipocytes from tumor-bearing and tumor-free breasts were collected and their expression profiles were analyzed. In response to tumor, adipocytes have unique expression patterns and may bring a tumor-permissive environment. Bergqvist et al. analysed adipocyte secretome under normal and obese-like conditions and found that adipocytes promote breast cancer proliferation through adipokine receptor CAP1. In addition to mature adipocytes, adipose stem cells also play a role in the interaction with tumor cells. Fajka-Boja et al. identified that a subpopulation of adipose stem cells, polyploid adipose stem cells, could promote breast cancer cell proliferation. Wu et al. discussed the interesting question of how senescence of CAA affects tumor progression. These research findings provide mechanistic insights into direct adipocyte-tumor cell effects. Other than that, indirect pro-tumor effects have also been described in the current topic. Delort et al. found that adipocytes enhance tumor invasion by altering the phenotype of a less studied mammary cell type, breast myoepithelial cells. Yadav et al. showed that adipocytes educate inflammatory cells, which consequently enhance angiogenesis, a critical process for tumor development and metastasis.

Besides breast cancer, contributors have made clinically relevant explorations in colorectal and pancreatic cancers, which are closely associated with adipocytes. Abu et al. reported that CRC patients with high BMI were able to release extracellular vesicles with immunoregulatory functions. Cheng et al. examined the role of immune signaling in colorectal cancer metastasis. Yang et al. explored therapeutic targets for pancreatic cancer. Generally, these manuscripts provide new evidence of adipocyte-tumor cell crosstalk in the tumor microenvironment.

In the tumor macroenvironment, several works in the current topic discussed the communication between tumor and adipose tissue in the aspect of metabolic homeostasis and cancer-associated complications. Sun et al. discussed the details of adipose tissue wasting in CAC development and summarized CAC animal models. Trivanović et al. briefly discussed the adipocyte-tumor crosstalk. Distant adipose tissues promote tumor development by releasing regulatory factors into the circulation. Wang et al. found that adipose-derived mesenchymal stem cells promote tumor progression *via* exosomes. Adipose tissue might communicate with cancer *via* metabolites. Recent progress in our understanding of cancer metabolism has made metabolism one of the most exciting areas of tumor biology and tumor therapy. Ye et al. summarized obesity-related metabolic alterations in cancer-associated host cells in tumors and discussed how these alterations foster tumor development. Together, we present multiple articles that focus on remote organ-organ communications in the context of cancer, which is not fully studied.

Adipose tissues have interesting and diverse functions in regulating tumor progression. This topic provides some advances and new perspectives on this field. It should be noted that the type of adipose tissue covered in this topic is mainly white adipose tissue. The crosstalk between brown adipose tissue and tumor needs to be further explored (Seki et al., 2022). We thank the contributing authors and reviewers for this topic. The increasing knowledge in the field is contributing to our understanding of the complex adipocyte-tumor cell crosstalks and to the discovery of novel cancer therapeutic options.

## References

[B1] Al-Zhoughbi W. Huang W. Paramasivan J. Till G. S. Pichler H. Guertl-Lackner M. (2014). Tumor macroenvironment and metabolism. Seminars Oncol. 41 (2), 281–295. Epub 2014/05/03PubMed PMID: 24787299; PubMed Central PMCID: PMCPMC4012137. 10.1053/j.seminoncol.2014.02.005 PMC4012137 24787299

[B2] Baracos V. E. Martin L. Korc M. Guttridge D. C. Fearon K. C. H. (2018). Cancer-associated cachexia. Nat. Rev. Dis. Prim. 4, 17105. Epub 2018/01/19PubMed PMID: 29345251. 10.1038/nrdp.2017.105 29345251

[B3] Bochet L. Lehuédé C. Dauvillier S. Wang Y. Y. Dirat B. Laurent V. (2013). Adipocyte-derived fibroblasts promote tumor progression and contribute to the desmoplastic reaction in breast cancer. Cancer Res. 73 (18), 5657–5668. Epub 2013/08/02PubMed PMID: 23903958. 10.1158/0008-5472.CAN-13-0530 23903958

[B4] Hoy A. J. Balaban S. Saunders D. N. (2017). Adipocyte-tumor cell metabolic crosstalk in breast cancer. Trends Mol. Med. 23 (5), 381–392. Epub 2017/03/24PubMed PMID: 28330687. 10.1016/j.molmed.2017.02.009 28330687

[B5] Iwamoto H. Abe M. Yang Y. Cui D. Seki T. Nakamura M. (2018). Cancer lipid metabolism confers antiangiogenic drug resistance. Cell. Metab. 28 (1), 104–117. e5. Epub 2018/06/05PubMed PMID: 29861385. 10.1016/j.cmet.2018.05.005 29861385

[B6] Jafari N. Kolla M. Meshulam T. Shafran J. S. Qiu Y. Casey A. N. (2021). Adipocyte-derived exosomes may promote breast cancer progression in type 2 diabetes. Sci. Signal. 14 (710), eabj2807. Epub 2021/11/24PubMed PMID: 34813359; PubMed Central PMCID: PMCPMC8765301. 10.1126/scisignal.abj2807 34813359 PMC8765301

[B7] Quail D. F. Dannenberg A. J. (2019). The obese adipose tissue microenvironment in cancer development and progression. Nat. Rev. Endocrinol. 15 (3), 139–154. Epub 2018/11/22PubMed PMID: 30459447; PubMed Central PMCID: PMCPMC6374176. 10.1038/s41574-018-0126-x 30459447 PMC6374176

[B8] Seki T. Yang Y. Sun X. Lim S. Xie S. Guo Z. (2022). Brown-fat-mediated tumour suppression by cold-altered global metabolism. Nature 608 (7922), 421–428. Epub 2022/08/04PubMed PMID: 35922508; PubMed Central PMCID: PMCPMC9365697. 10.1038/s41586-022-05030-3 35922508 PMC9365697

[B9] Sheng X. Parmentier J. H. Tucci J. Pei H. Cortez-Toledo O. Dieli-Conwright C. M. (2017). Adipocytes sequester and metabolize the chemotherapeutic daunorubicin. Mol. Cancer Res. 15 (12), 1704–1713. Epub 2017/11/10PubMed PMID: 29117945; PubMed Central PMCID: PMCPMC5726435. 10.1158/1541-7786.MCR-17-0338 29117945 PMC5726435

[B10] Song Y. C. Lee S. E. Jin Y. Park H. W. Chun K. H. Lee H. W. (2020). Classifying the linkage between adipose tissue inflammation and tumor growth through cancer-associated adipocytes. Mol. Cells 43 (9), 763–773. Epub 2020/08/08PubMed PMID: 32759466; PubMed Central PMCID: PMCPMC7528682. 10.14348/molcells.2020.0118 32759466 PMC7528682

[B11] Yang Y. Cao Y. (2022). The impact of VEGF on cancer metastasis and systemic disease. Seminars Cancer Biol. 37, 289. Epub 2022/03/22PubMed PMID: 35307547. 10.1016/j.semcancer.2022.03.011 35307547

